# Cutaneous metastases from esophageal adenocarcinoma: Taking clues from dermoscopy

**DOI:** 10.1016/j.jdcr.2025.05.013

**Published:** 2025-06-10

**Authors:** Bailey Patrick, Anne-Taylor Beck, Anna Ballenger

**Affiliations:** a University of Missouri School of Medicine, Columbia, Missouri; b Department of Dermatology, University of Missouri Health Care, Columbia, Missouri

**Keywords:** adenocarcinoma, dermatology, dermoscopy, neoplasia

## Clinical presentation

**Fig 1:**
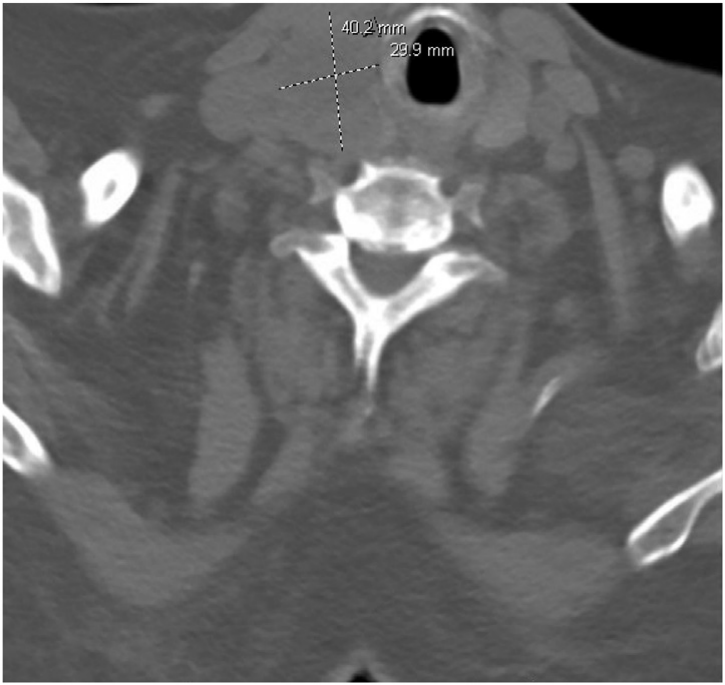
Computed tomography (CT) scan showing the esophageal adenocarcinoma.

An 81-year-old male presented to the emergency department for altered mental status with dysarthria and memory loss. Brain magnetic resonance imaging demonstrated acute lacunar infarcts consistent with ischemic stroke. Additional imaging revealed widespread metastatic disease with an esophageal mass thought to be the primary (Fig 1). A biopsy of the distal esophageal mass was performed which showed invasive moderately-differentiated adenocarcinoma in the context of gastroesophageal reflux disease.

**Fig 2:**
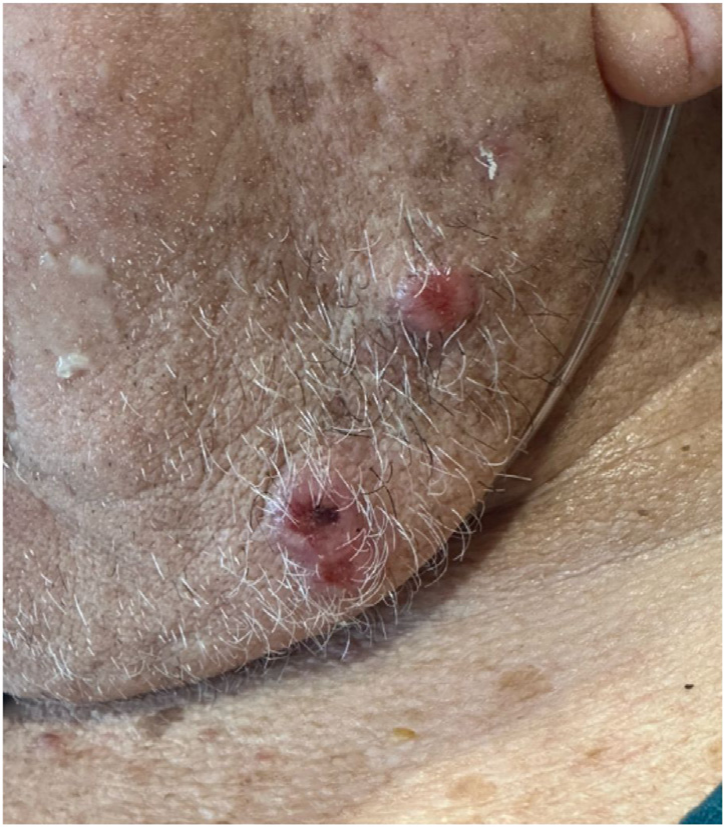
Gross photograph of cutaneous metastases of esophageal adenocarcinoma to the face.

**Fig 3:**
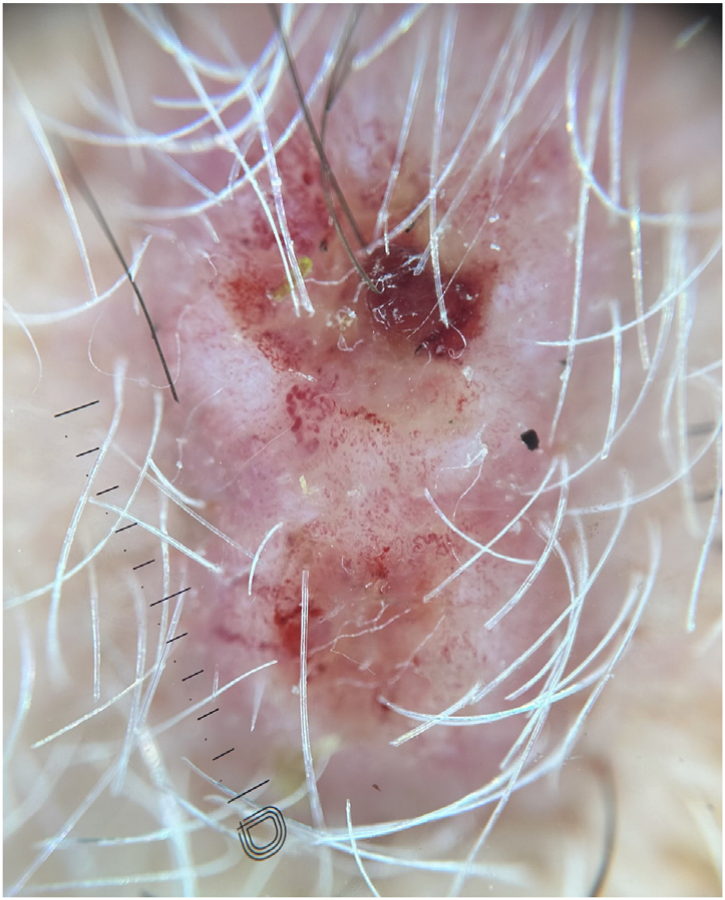
Dermoscopy of cutaneous metastasis of esophageal adenocarcinoma with notable peripheral coiled vessels.

**Fig 4:**
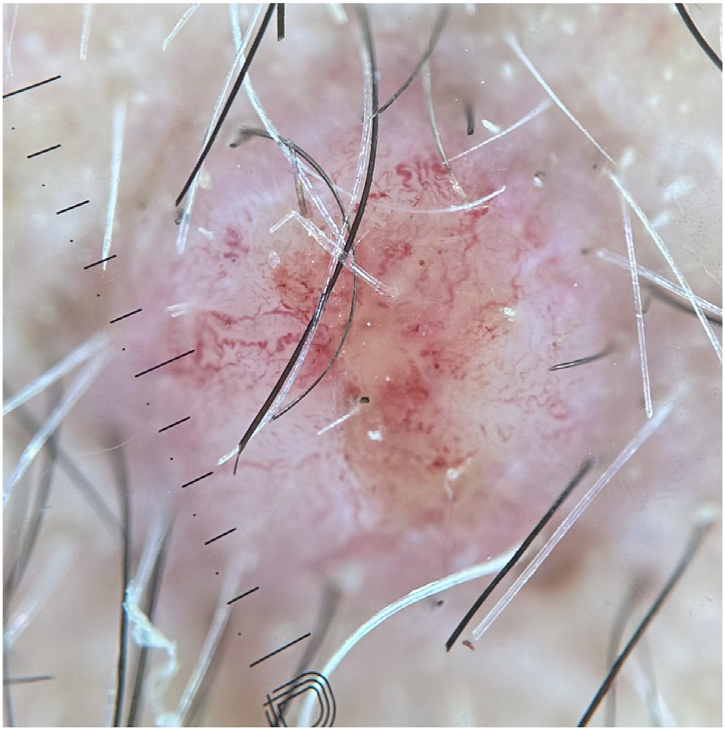
Dermoscopy of cutaneous metastasis of esophageal adenocarcinoma with notable serpentine vessels.

In addition to altered mentation, the patient’s family reported rapid development of 3 hemorrhagic crusted nodules along the left jawline (Fig 2). On examination, he was found to have 3 discrete nodules on the left jawline and an additional smaller similar papule on the right upper chest. Dermoscopy of these lesions was notable for large caliber coiled and serpentine vessels with a central white amorphous structureless region (Fig 3 and 4). Although cutaneous metastases are rare, current literature states that these lesions are typically red or pink nodules with prominent vessels on dermoscopy.^1^^,^^2^

One of the nodules on the left jawline was biopsied. The specimen demonstrated infiltration of atypical pleomorphic glands with prominent nucleoli and vacuolated cytoplasm along with dermal necrosis consistent with cutaneous metastasis of adenocarcinoma. This case illustrates the unique and striking dermoscopic features of cutaneous metastasis of adenocarcinoma. Lesions with these features in a patient with known malignancy should raise suspicion for metastasis to the skin. Ultimately, given his functional status and several comorbidities, the patient chose hospice care.

## Conflicts of interest

None disclosed.
